# The CELLmicrocosmos Tools: A Small History of Java-Based Cell and Membrane Modelling Open Source Software Development

**DOI:** 10.1515/jib-2019-0057

**Published:** 2019-09-27

**Authors:** Bjorn Sommer

**Affiliations:** Royal College of Art, School of Design, Innovation Design Engineering, London SW7 2EU, UK

**Keywords:** Integrative Bioinformatics, Cell Modelling, Molecular Modelling, Java, Open Source Software

## Abstract

For more than one decade, CELLmicrocosmos tools are being developed. Here, we discus some of the technical and administrative hurdles to keep a software suite running so many years. The tools were being developed during a number of student projects and theses, whereas main developers refactored and maintained the code over the years. The focus of this publication is laid on two Java-based Open Source Software frameworks. Firstly, the CellExplorer with the PathwayIntegration combines the mesoscopic and the functional level by mapping biological networks onto cell components using database integration. Secondly, the MembraneEditor enables users to generate membranes of different lipid and protein compositions using the PDB format. Technicalities will be discussed as well as the historical development of these tools with a special focus on group-based development. In this way, university-associated developers of Integrative Bioinformatics applications should be inspired to go similar ways. All tools discussed in this publication can be downloaded and installed from https://www.CELLmicrocosmos.org.

## 1 Introduction

*CELLmicrocosmos (Cm)* is a cell modelling and visualization project with roots going back to the year 2003 when a first cell animation in the context of a Bachelor thesis – in Media Informatics and Design at Bielefeld University – lay the basis for the follow-up projects [1]. Starting from an early cell visualization prototype which was developed with commercial software, the decision was made to move away from proprietary software towards Open Source software. This decision enabled us to advance the tools over more than one decade at now three institutions with the fourth one right here to take over. Thanks to the work of many students – which were directly or indirectly involved by doing their bachelor, master or diploma projects or theses – the tools were advanced by modelling and designing, system engineering and coding, authoring, testing and evaluating. In 2012, a PhD thesis discussing a large part of the historical development was published [2].

Here, the focus is laid on the software development part, more precisely on the two major Integrative Bioinformatics tools which were being developed in the context of Cm and which are still being maintained, used and cited in the current time: the Java-based *CELLmicrocosmos CellExplorer with the PathwayIntegration (CmPI)* and the *CELLmicrocosmos MembraneEditor (CmME).* In the context of this project, Integrative Bioinformatics can be understood as the combination of data science with modelling and visualization techniques for the purpose of analyzing biological systems – requiring the integration of many different data sources.

Of course, during the recent years, a number of other membrane and cell modelling approaches have been developed and published. Here, a short review of related work for both major tools will follow. Moreover, tools should be mentioned which inspired the development of the CELLmicrocosmos tools from the start.

### 1.1 Related Membrane Modelling Tools

Until today, there are not many alternatives available for CmME. However, a number of approaches are discussed in the Membrane Packing Problems paper [3]. Nowadays, two tools gained a lot of interest which still are quite different from CmME.

The command line-driven tool *PackMol* is a very popular tool for creating molecular structures which is also based on geometry-based packing algorithms. It enables the definition of different assemblies by using scripts. In contrast to CmME, the original version does not provide a user interface and no viewer which could be used to directly access the molecular structure [4].

The web-based tool *CHARMM-GUI Membrane Builder* can be used to create small membrane patches or even vesicles (which are nowadays also supported by the Vesicle Builder plugin for CmME). For this purpose, it is making use of the replacement and insertion method using partly pre-generated simulated membrane models. A big advantage is the large number of pre-generated membranes here. On one hand, the user can easily use the server capabilities to create the membrane models, on the other hand the user relies on the limitations of the server capabilities. CmME has the advantage that the user can use basically every PDB file as the base for the membrane generation process [5], [6].

A very good recent overview in terms of other membrane modelling and visualization approaches is provided by [7].

### 1.2 Related Cell Modelling Tools

Only a few mesoscopic modelling and visualization tools are available. The following tools where developed after the first version of the CellExplorer was released:

cellPACK represents an approach to fill cells with shapes generated from PDB files by using so-called recipes – distribution algorithms applied to whole-cell environments [8]. The so-generated cells are basically intended for illustrative purposes and cannot be used for simulations. cellVIEW was recently created to create and visualize cellPACK-based by making use of GPU programming [9].

A web tool to explore microscopy-based 3D models is the Allen Cell Explorer providing whole cell maps of microscopic data [10].

A tool which inspired the development of CmPI was MetNet 3D and VR which combined metabolic pathways with a cell environment [11], [12]. However, this cell environment was very abstract and just used cubes as cell component representatives.

On the other hand, there was a tool which was able to create 3D metabolic networks creating VRML files compatible to web browsers – this tool was very interesting as motivation to create 3D biological networks [13].

### 1.3 Content of this Publication

The major purpose of this publication is (1) to discuss the quite diverse routes of development the two tools had to take and (2) to show some technical details which were so far not discussed in our previous publications and which helped us to maintain these projects over so many years.

First, the developmental history of the different projects will be discussed in Chapter 2, Developmental History – The Evolution of CELLmicrocosmos tools. In Chapter 3, Overview of CELLmicrocosmos Java-based Projects, the purpose and the main features of the different projects will be introduced. Then, in Chapter 4, Technology & Implementation, the architecture, implementation and workflows of the different projects will be discussed. Chapter 5, Results and Discussion, provides an overview of outcomes generated with the different tools, and Chapter 6, Outlook, concludes with the future perspectives for the different tools.

## 2 Developmental History – The Evolution of CELLmicrocosmos Tools

The two aforementioned major projects were not part of an initial plan – they emerged over the years of development. Moreover, the development of the new projects took two quite different routes: while most of the development of CmME was basically developed by a very small core team and had over many years only a single main developer, CmPI integrates a large number of different student projects realized by a number of individuals.

**Figure 1:**
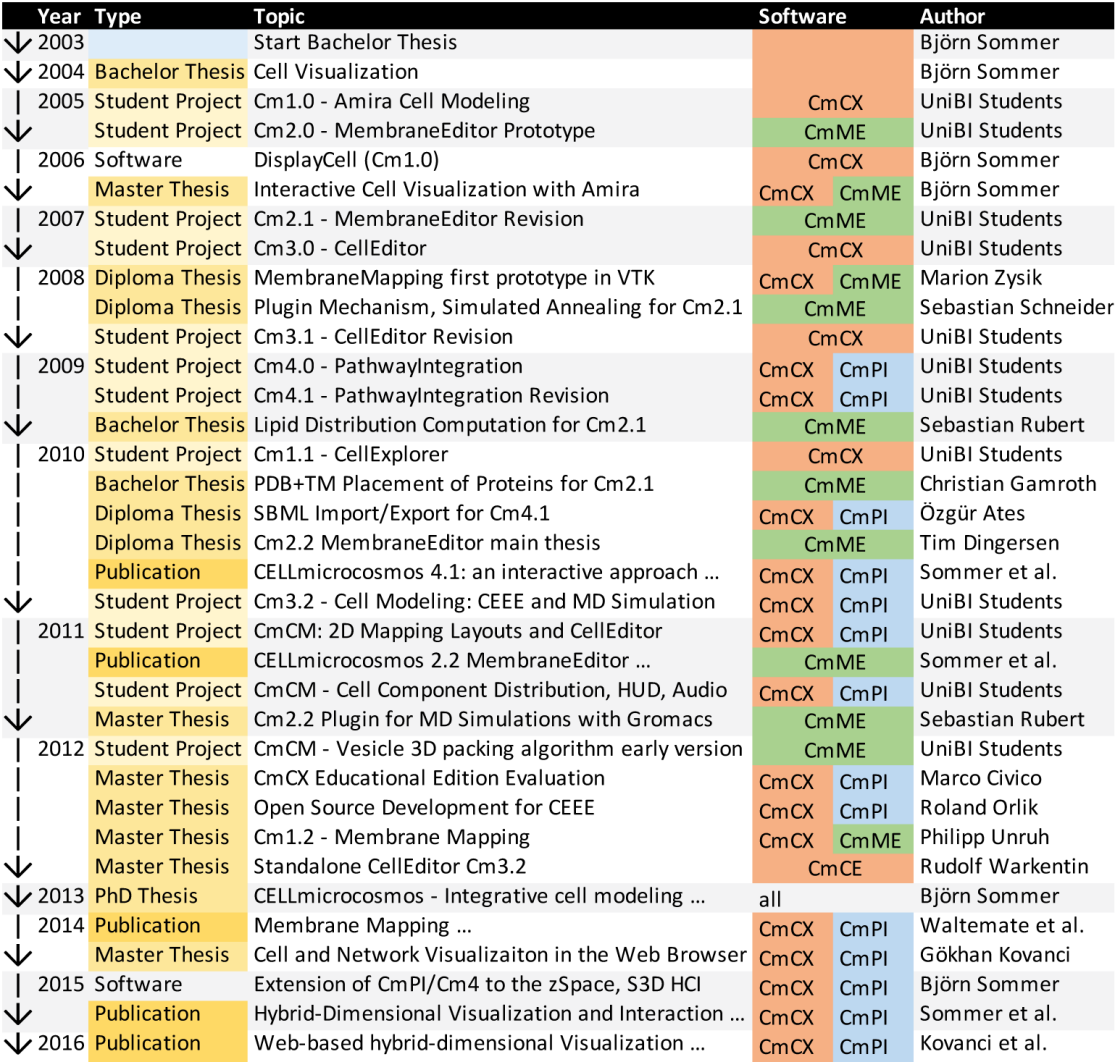
An overview of core software developments discussed here and their context.

**Figure 2:**
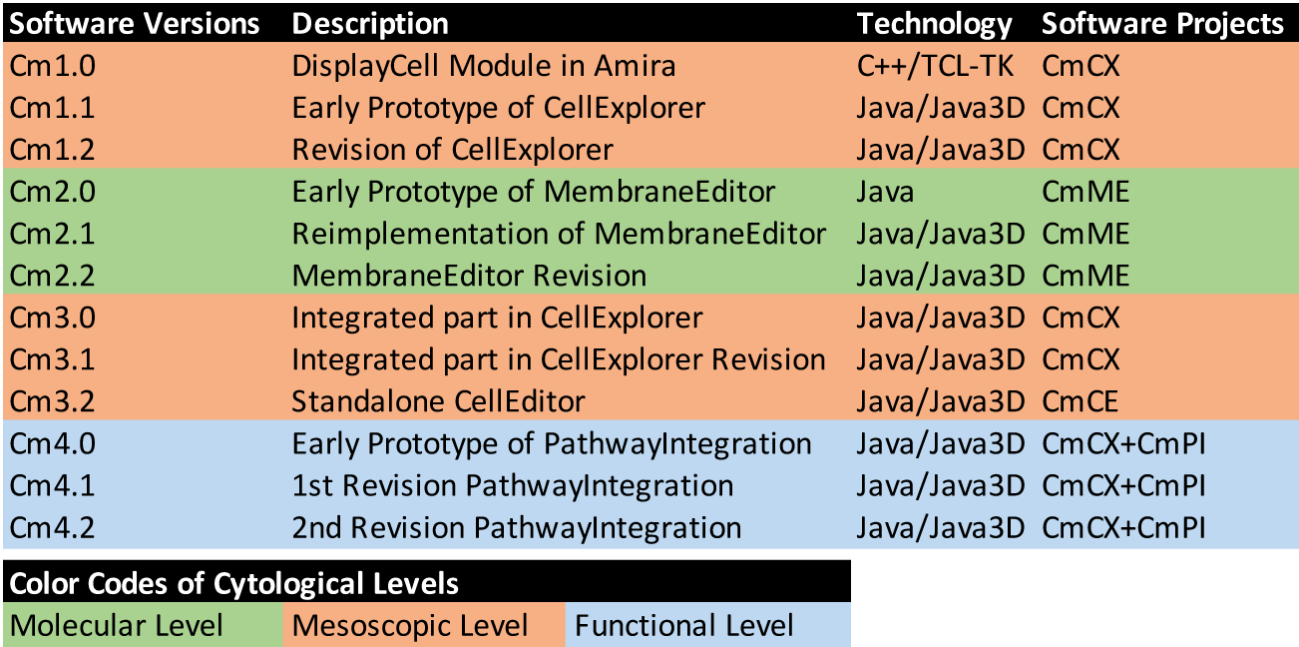
An overview of software versioning of the CELLmicrocosmos project (top) and the colour coding of the Cytological Levels (bottom).

Figure 1 shows an overview of the developments to be discussed in the following chapters. It focuses on the years from the start of the CELLmicrocosmos project 2003 to the publication of the PhD thesis and two more recent developments around 2015–2016. Since then, the software is still being extended and updated, but the major version number was not changing. The colour coding of the software is based on the three cytological levels: red – mesoscopic level, green – molecular level, blue – functional level, as shown in Figure 2, bottom.

Figure 2 shows that the first number of the different software projects indicates the project number: Cm1 – CellExplorer, Cm2 – MembraneEditor, Cm3 – CellEditor, and Cm4 – PathwayIntegrations. The software versions X.0 in Figure 2 were basically a sketch of the software to be developed. The first iteration, X.1, was then a completely new project (Cm1 and Cm2) or an extensive extension and refactoring of the previous version (Cm3 and Cm4). In case of Cm3, the version Cm3.2 was the first standalone version of the *CellEditor (CmCE).* It was based on code part of CmCX but drastically extended as it was decided that the functionality goes far beyond the one to be expected of an explorative software. Please also note that the software versions number are only partly connected to the version numbers used in the student software projects as shown in Figure 2: For Cm2, the software versions match the student project versions, Cm2.0 and Cm2.1, but then version Cm2.2 was developed during a diploma thesis. But e.g. the student project Cm3.2 was pushing forward the CmME as well as CmPI project, plus the initial version of the standalone Cm3.2 CellEditor (CmCE).

### 2.1 CELLmicrocosmos DisplayCell – Our First Prototype of a CellExplorer Developed with Amira

**Figure 3:**
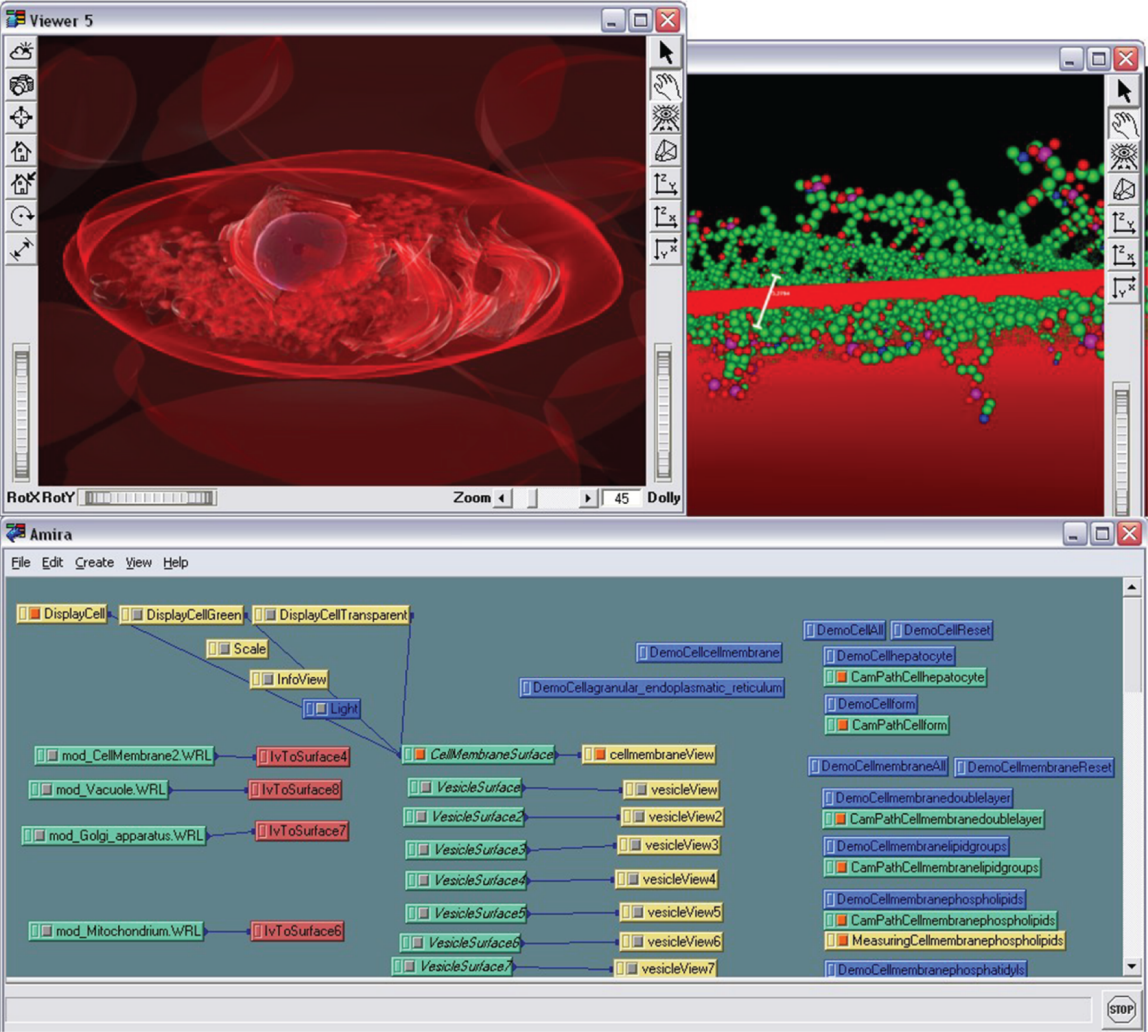
CELLmicrocosmos DisplayCell in Amira: The first software prototype of the CmCX: top left – the mesoscopic perspective of a hepatocyte cell; top right – the molecular perspective showing the membrane manually attached to the surface of the membrane; bottom: the modular structure of the environment, consisting of different cell component surfaces, (camera) animations, labels, light sources, etc.

While the previously mentioned Bachelor thesis did not provide a software tool but instead an animation and a number of 3D cell component models (such as a cell membrane, mitochondrion, etc.) which were used with the different tools in the following years, a Master thesis created a first prototype which was able to show a cell model in 2006 [14]. Here, an educational mesoscopic cell model was created (Figure 3 top left) which already manually attached an molecular/atomistic membrane to the surface of 3D mesh of a cell membrane (Figure 3 top right), a process which we have later automatized and published as Membrane Mapping [15]. Membrane Mapping is a technique which is today also found in cell visualization tools, such as cellPACK or cellVIEW [8], [9]. This software sketch was realized with Amira 3 and Mercury Amira 4^®^, developed at the Zuse Institut Berlin by the group of Hans-Christian Hege, nowadays commercially distributed by Thermo Fisher Scientific^®^ [16], [17]. Amira is usually a tool which can play a significant role in a cell visualization pipeline, as it provides segmentation capabilities for microscopic images. Related Open Source tools are ImageJ/Fiji or ICY [18], [19]. Also in the context of Cm, some of these tools play an important role which will not be discussed here any further – the interested reader is referred to [20].

Amira has a modular structure and also the internal combination of different modules (Figure 3 bottom) played an important role for our later projects. Moreover, Amira was already able to make use of our semi-immersive back projection system which we used at this time at Bielefeld University, and in this way enabled us to present the 3D cell model in stereoscopic 3D.

The lessons learnt from this early prototype were:

– We will need an Open Source approach to guaranty future development without incalculable costs, especially in terms of publishing the software later on.
– We need a tool to generate the molecular heterogeneous membrane, as we did not find an appropriate tool able to do so.
– For exploring cells, a simple-to-use navigation is required which is not provided by Amira.

### 2.2 CELLmicrocosmos MembraneEditor – from a first Java-Based Prototype to a Java Software Suite

The problem of generating a membrane in the context of Cm was directly addressed by starting a first student project in winter term 2005/2006. A simple Java tool was developed which was able to distribute provided PDB files of lipid models onto a membrane with a very simple linear placing algorithm. This project was later known as CELLmicrocosmos 2.0 and was officially led by my first supervisor of my Bachelor as well as Master thesis, Dr. Dieter Lorenz. While transforming later to the project leader and educator, I was here one of the group participants and programmers – although already providing the basic requirements for the tool to be developed.

**Figure 4:**
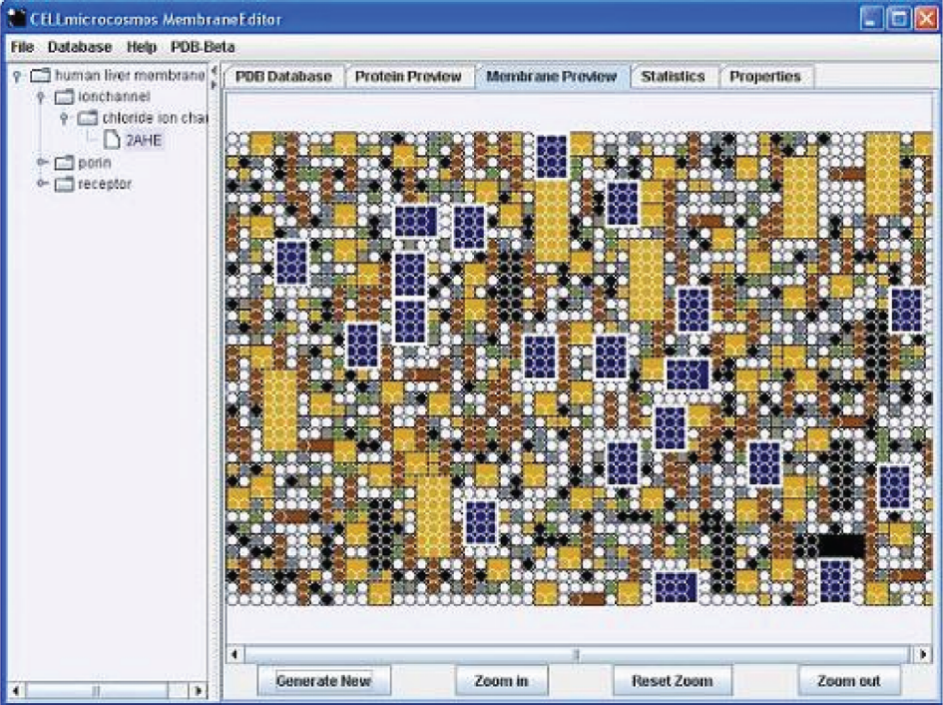
CELLmicrocosmos 2.0: The first version of the MembraneEditor.

Figure 4 shows this early prototype – the visualization was extremely simple and was only shown in 2D. But proteins could be already manually aligned to the membrane by using the externally developed Jmol tool which is also based on Java [21].

The success of the project was that I was able to use a membrane generated here already in my Master thesis from 2006. But in the winter term 2006/2007 I was leading the follow up project 2.1 and it was decided to create a new tool from scratch. Six students were developing this tool, but during the process a lead programmer emerged who later took over the main development during his diploma thesis and in the context of his student job: Tim Dingersen. His development was accompanied by Sebastian Schneider who was developing a relatively complex simulated annealing algorithm for the MembraneEditor.

Therefore, a very effective small team was driving the development, and a very important factor during this time was that I was regularly attending related conferences – such as the German Conference on Bioinformatics or the Molecular Modelling Workshop in Erlangen – and in this way was able to talk to many researchers during our poster presentations and get feedback regarding requirements on our MembraneEditor (e.g. [22], [23]). These ideas were directly injected in the development process and enabled us to create a quite comfortable membrane modelling tool which is still unique in its functionality until today.

The first major publication of the MembraneEditor is up to date the most cited paper of the Cm project and shows that it was integral part of a number of papers creating initial structures for molecular dynamic simulations [24].

Another important aspect of the MembraneEditor was the development of a plugin interface which was later also adapted by the CellExplorer project. This plugin interface enabled students who are not familiar with the complex source code of the MembraneEditor to develop their custom algorithms by using the corresponding interface.

Following from this, CmME has three application areas:

– setup of initial structures for molecular simulations,
– modelling of membranes for illustrative purpose,
– modelling of membranes for testing initial properties of the membrane, such as area per lipid or thickness with other tools [25], and
– development of new Membrane Packing Algorithms.

### 2.3 CELLmicrocosmos PathwayIntegration – Evolution from a Java-Based Prototype to a Tool for Multiple Purposes

To avoid confusions right from the start: the CELLmicrocosmos CellExplorer (CmCX) is the basic framework which has one module called the PathwayIntegration (CmPI). The first digital sketch for the CellExplorer was indeed the initially discussed DisplayCell realized with Amira. In the years 2007–2009, five student projects took place at Bielefeld University which were all dedicated to the development of this tool.

The big difference in comparison to the development of CmME was the fact that each project builds directly on top of each other. While CmME had over a long period only a single main developer, I had to take over the role of the lead developer quite early for this project – but my major task was to integrate the large amount of different codes from different students into one large project. Of course, I had to refactor the project from time to time, and as the PathwayIntegration module was a major part of my PhD thesis, the relation student code to my code might be 1:10 in this module, but basically up to today from each involved student code snippets will be found in the CellExplorer.

Ober the years, a number of application cases emerged for CmCX:

– setup of simple cell models which can be used as a base for cell visualizations with external tools like Blender,
– visual subcellular protein localization by using database-derived information and mapping of protein-related networks onto cell components with the internal CmPI module,
– educational cell exploration by combining the cell model with Wiki-like information, and
– hybrid-dimensional large-scale cell exploration in Virtual Reality with the zSpace 200^®^ and the CAVE 2^®^ at Monash University [26].

### 2.4 Different Places, Different Faces – Same Tools

A large part of the software development was done during the years 2005–2015 while my time as a graduate student and later as research assistant and PostDoc at Bielefeld University in the Bio-/Medical Informatics Group of Ralf Hofestädt.

2015 to 2016 the project – especially the CellExplorer – was advanced at Monash University in Melbourne during my time as research fellow in the group of Falk Schreiber. Here, the CAVE 2^®^, a large multi-screen display environment was used to navigate cell models created in the CellExplorer by using a zSpace 200^®^ [26].

From 2016 to 2019 the project was maintained while I was PostDoc at the University of Konstanz in the Life Science Informatics group of Falk Schreiber. Here, CmME was used as part of a membrane simulation project whereas CmCX was combined with VANTED [27], [28], [29].

While the tool is still being used at the University of Konstanz, the development is now transitioning to the Royal College of Art in London (as of 2019).

## 3 Overview of CELLmicrocosmos Java-Based Projects

We discussed now, how the different models were created. Whereas CmPI was going through many stages of refactoring and was transformed over years from a quick prototype with repetitive code to a software suite, CmME had – as the new version was started from scratch – a relatively clear structure. Both software architectures are based on the Model-View-Controller principle.

In the following, the two major frameworks will be quickly introduced:

### 3.1 CELLmicrocosmos MembraneEditor (CmME)

CmME provides membrane modelling based on the PDB format [30]. It has a number of features which are in this combination unique [24]:

– Lipid composition based on absolute numbers or percentages to create heterogeneous membranes,
– WYSIWYG modelling of membranes with drag’n’drop functionality,
– Shape-based visualization as well as atom-based visualization of atomic structures,
– Modelling of different membrane layers to, e.g. stack membranes or create different vesicle-like structures,
– Drawing of microdomains of differing lipid compositions from the environment to create, e.g. lipid rafts or different shapes,
– Semi-automatic placement of proteins based on the OPM database and the PDB_TM database to align proteins with the membrane normal [31], [32],
– Direct download from the PDB database giving access to all published PDB files,
– Extensive PDB format export settings enabling compatibility with basically all tools supporting PDB format, and
– Membrane Packing Algorithm interface to import and compile algorithms on the run and supporting reproducible membrane generation.

There are basically three application cases for CmME:

– Straight forward modelling of heterogeneous membranes for visualization purposes,
– Validation of membrane compositions regarding density/area per lipid with, e.g. APL@Voro [25], and
– Creation of starting configurations for molecular simulation tools like Gromacs [33].

**Figure 5:**
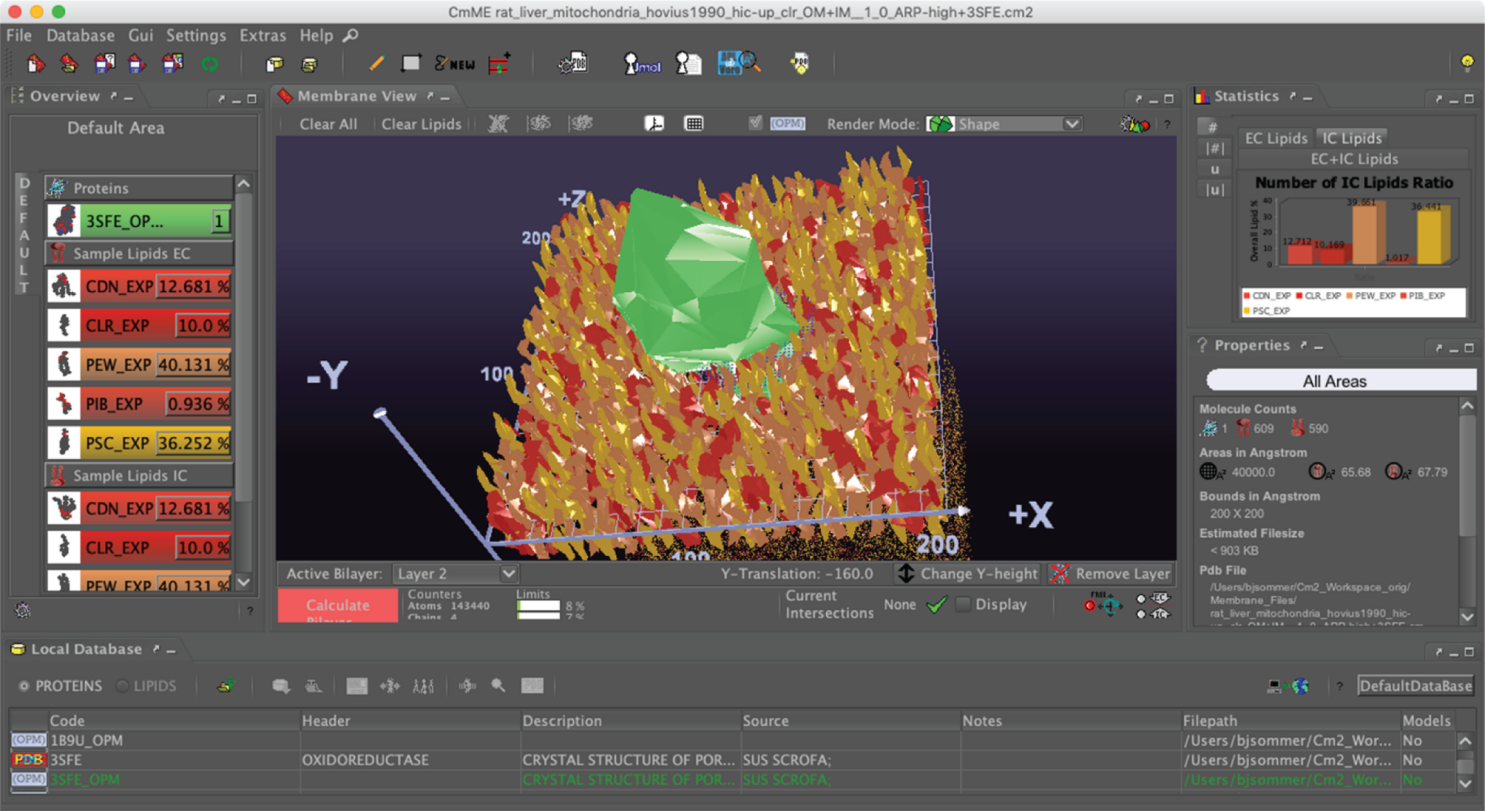
CELLmicrocosmos MembraneEditor: Top Left – The five lipid types are indicated by different colours – the corresponding initial percent ratios are shown at the left side; Top Right – the resulting percent ratios after generating the membrane; Bottom Right – information about the actual membrane model is indicated, as are data on the average lipid density and the molecule numbers; Top Center – the Membrane View; Bottom – the database containing different PDB lipid or protein models.

Figure 5 shows the GUI with the MembraneEditor with the following basic windows:

– Overview: Membrane composition with proteins and lipids incl. percentages added to the membrane model,
– Local Database: the database containing the protein and lipid models,
– Properties: the properties of the membrane, such as overall lipids per membrane, proteins inside the membrane, etc.,
– Statistics: the effective lipid percentages after the membrane was generated by using one of the Lipid Packing Algorithms,
– Membrane View: the 3D view of the membrane showing the shape-based visualization of molecules: the lipids surround a single protein in the centre of the 200 × 200 Å^2^ membrane,
– The red button starts the membrane packing process.

The application incl. videos and documentation can be found at: http://Cm2.CELLmicrocosmos.org.

### 3.2 CELLmicrocosmos CellExplorer (CmCX) with the PathwayIntegration (CmPI)

The CellExplorer with the PathwayIntegration supports the stereoscopic 3D exploration of cells, providing different modular eukaryotic and prokaryotic cell models. The following features are part of this software:

– Import and export of cell models in VRML97/2.0 format, making it compatible with many modelling packages, such as Blender or Autodesk 3ds max^®^.
– Exploration of cell models with three different navigation modes providing a 6DOF (six degrees of freedom) navigation, enabling the user to move around cell component models or traveling across them [34],
– Connection to the Bio Data Warehouse (BioDWH) to localize proteins/genes (e.g. to the inner membrane of a mitochondrion or the cell membrane), containing a number of databases, such as UniProt, Brenda, and Gene Ontology, and for text mining-related data, ANDCell [35], [36], [37], [38],
– 2D Network Visualization based on the JUNG library, visualizing, e.g. metabolic or protein-protein interaction networks [39],
– Different Node Placing algorithms to apply reproducible 3D network layouts to cell environments, mapping the networks onto the 3D cell components based on the database-derived protein/gene localizations,
– 3D Stereoscopic Visualization featuring position-based adjustment of the stereo vision in case compatible hardware, such as a 3D TV is used [40].

**Figure 6:**
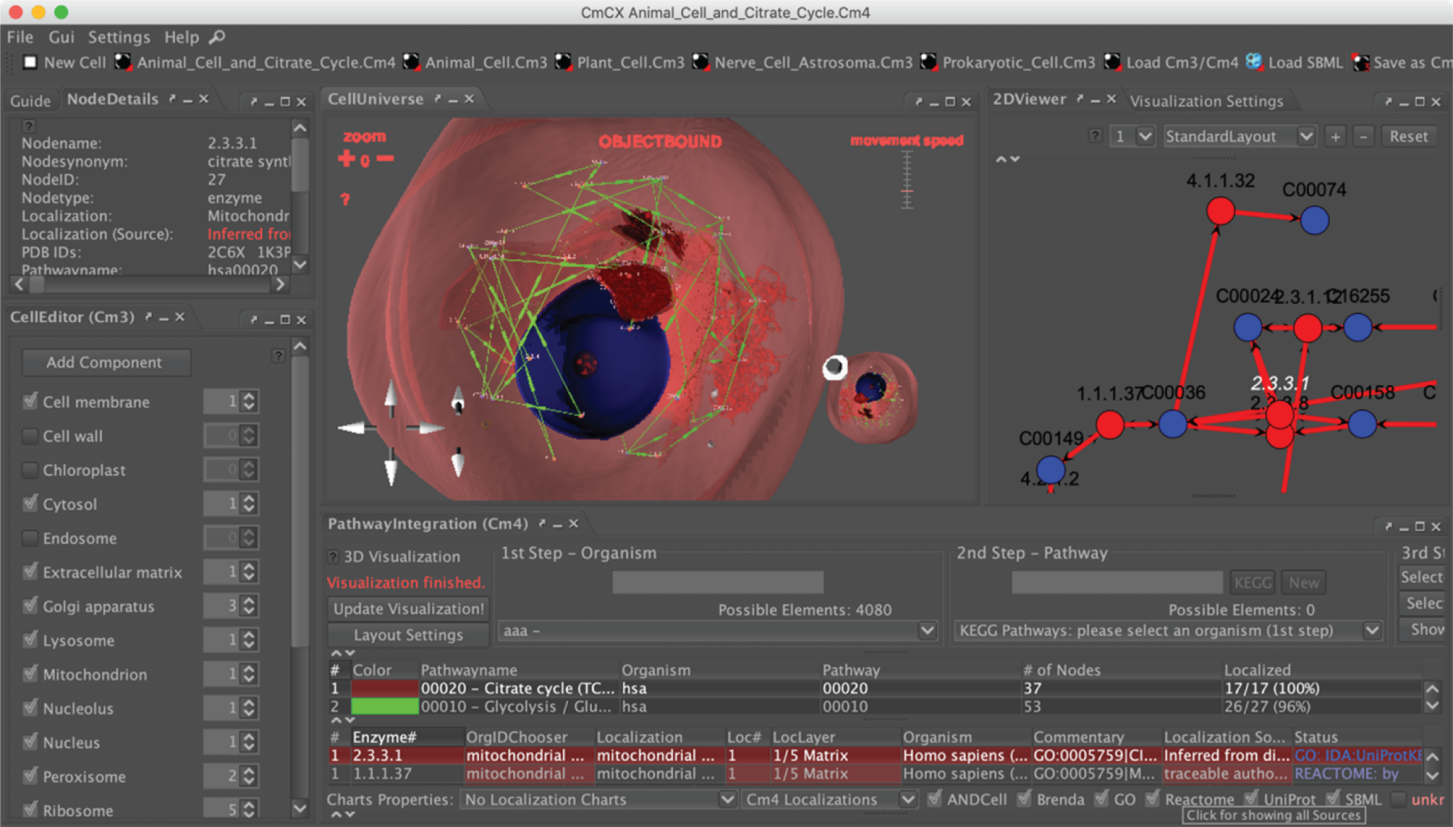
CELLmicrocosmos CellExplorer with the PathwayIntegration: Bottom Right – PathwayIntegration Window; Left – CellEditor window; Top Center – 3D view of the cell; Top Right – the metabolic pathway as downloaded from KEGG.

The following basic windows are part of the application:

– CellEditor (CELLmicrocosmos 3.1): a simple interface to add new cell component models to the cell environment which can be used to add new cell components and manipulate their position with mouse and keyboard,
– PathwayIntegration (CELLmicrocosmos 4.2): the pathway (Figure 6 top) and protein localization table (Figure 6 bottom) which can be used to download KEGG pathways and protein localization from BioDWH,
– 2DViewer: 2D network visualization using the JUNG library (Figure 6 top right),
– CellUniverse: 3D visualization of the cell (Figure 6 top),
– NodeDetails: information about the currently selected protein (Figure 6 top left).

The tool can be found at: http://Cm4.CELLmicrocosmos.org.

A derivate of this project is the CELLmicrocosmos 3.2 CellEditor/CmCE. The whole software is based on the CellExplorer which – like previously mentioned – already contains a simple CellEditor (version 3.1). We decided that the functionality of the CellEditor had to be extended in a way going far beyond the scope of the CellExplorer. Therefore, a separate software was developed which is able to create cell models which can be directly imported into CmCX/CmPI. This CellEditor is able to work with different VRML97 formats and save same in a CmCX-compatible format. However, the software was only used for internal purposes so far and has been not released by now. http://Cm3.CELLmicrocosmos.org.

In addition, a strongly simplified web-based 3D visualization software exists based on Three.js and D3.js which can be used as an online viewer of localization scenarios of original files from CmPI [41]: http://Cm4web.CELLmicrocosmos.org.

## 4 Technology and Implementation

Now that a basic overview of the functionality was provided, the basic technology of the framework should be discussed.

### 4.1 The Backbone: Java

The first version of CmME was developed with Sun Java 6. The big advantage of Java is its cross-platform compatibility. First, the software was developed for Windows, then optimized to be used also with Linux, and finally also released for Mac OS X. The reason why it was not directly available for all platforms will be discussed in the next section. Today, it runs with Java 8 but of course also the support of future Java versions is planned.

As the recent versions of Java are planned to be released as commercial versions, the CELLmicrocosmos tools will in future be only optimized for freely available versions of Java.

Another big advantage of Java is that it scales with new hardware developments. To provide an example: in the beginning of the development of CmME, it ran on 32 bit systems and 256 MB RAM had to be sufficient. In this configuration, membranes with a size of 200 × 200 Å^2^ could not be visualized, as the amount of memory was too low. The memory management in Java can be optimized by changing the Heap space – which represents the amount of memory which can be reserved for the running Java application. Therefore, nowadays CmME can be started without problems with dedicated 8 GB Ram, e.g. using a laptop with 16 GB Ram. In this way, already vesicles with a diameter of 200 Å can be modelled and exported. Following from this, created membrane models scale with the factor of memory available.

### 4.2 Visualization with Java3D

While the porting of basic Java software to different operating systems is quite straight forward, the porting of CmME and CmPI were more complex, as the 3D visualization is driven by Java3D. During the time of the first CELLmicrocosmos Java3D-based applications, Java3D 1.5.1, and shortly later 1.5.2 were released. For different operating systems, different binary libraries which connected the 3D visualization of Java to the graphics. On Windows, DLL files were required for this purpose. Interestingly, it was possible to use Java3D with DirectX as well as OpenGL in those days. A huge problem was the port to Mac OS X, as Mac OS was equipped with an outdated Java3D version (1.3) which prevented the direct use of an actual Java3D version. To cope with this problem, a script was written to break the link to this library during the installation process and install the new one (after warning the user).

But after Oracle acquired Sun, the development of Java3D was completely stopped (originally, a version 1.6 was planned). Therefore, the problem arose that a number of software frameworks stopped working as Java3D was not supported anymore.

**Figure 7:**
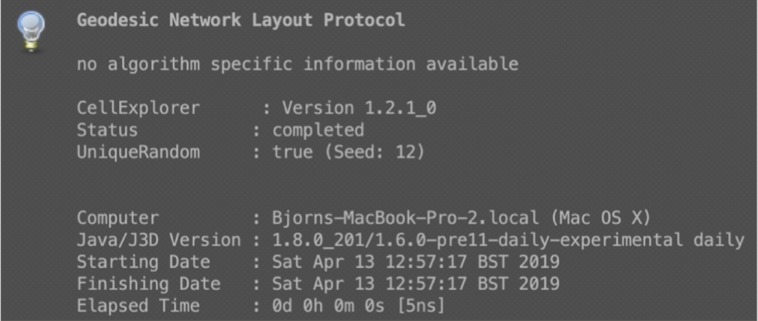
CELLmicrocosmos CellExplorer with the PathwayIntegration: The network layout protocol after associating a cell model with a metabolic network shows also the used version of Java as well as Java3D.

This is the reason why the JOGL developers decided to make a fresh port of Java3D which wraps the old functionality to their Java OpenGL framework. The big advantage coming with this change was that the application could also be exported to Mac OS X in much better quality, providing also better functionality. Nowadays, Java3D 1.6.2 is released and a pre-release of Java3D 1.7.0 exists. The current releases of CmCX as well as CmME currently run on Java3D 1.6.0, whereas first tests implementations with Java3D up to 1.7.0 were successful. The Java/Java3D versions are shown in both frameworks when a Node Placing Algorithm (CmPI) or a Membrane Packing Algorithm (CmME) were executed – see also Figure 7.

### 4.3 Java Web Start to Self-executable Jars

Quite early in the development Java Web Start technology was used to deploy both packages. Over the CELLmicrocosmos main webpage, the software suites could be directly downloaded, installed and started. Web Start enabled to start the tools offline and online. In case an online connection existed an online update of the packages was triggered. This mechanism still works up to this date, but Java Web Start is deprecated and will not be supported in future versions of Java (versions 9+). The Web Start mechanism worked especially very good on Windows- and Linux-based systems. On Mac OS X this approach was for a long period problematic because of the Java 3D problem discussed earlier.

Therefore, since a few years also self-executable packages are provided for the Cm project. These packages just require an installation of Java 8 on the local machine and can then be directly started. These packages however do not provide an update process.

In the future, alternative installation and update processes could be provided similar to those ones used by, e.g. Image/Fiji.

### 4.4 JavaScript with Three.js and D3.js

An alternative way to re-implement the applications discussed here would be to port them completely to browsers. This has partly been done with CmPI.

CmPIweb is based on Three.js and D3.js [41]. Three.js is based on WebGL, a subset of OpenGL compatible to all major browsers, such as Firefox, Chrome, Safari, etc. It provides a small subset of the functionality of CmPI. The basic intention is to visualize cell maps created with CmPI in the browser and provide basic navigation functionality. However, it is not possible to refine the network localization nor the cell models. An important feature is that a cell model can just be loaded and visualized by including the corresponding online location in the URL.

The biggest disadvantage of WebGL applications is that the browser naturally limits the use of memory. Especially for CmME, if large membranes with multiple millions of atoms are generated, this is a limiting factor. However, these problems could be solved by a complete reimplementation, as web-based molecular visualization tools, such as Mol*, provide approaches to visualize very large molecular structures [42].

### 4.5 Libraries and Licensing

As basically all large software frameworks, also CmPI and CmME integrate a number of external libraries. The libraries for CmCX, the basic cell visualization and modelling software, are shown in Table 1: JOGL’s Java3D are used for the 3D visualization, Substance and Infonode Docking Windows are used for the GUI and the Look and Feel, and JDOM for the XML parsing. Table 2 shows the extension of CmCX with the CmPI module: the JUNG library is used for the 2D network visualization, JFreeChart for the graph visualization, jSBML for the SBML import/export [43], and nearly all other libraries – such as Axis – are relevant for the connection to the MySQL database via Webservice. The membrane modelling and visualization tool CmME consists of the libraries shown in Table 3. Here, previously-mentioned libraries are used for similar purposes. New is Jmol which is used as an external library to efficiently visualize the atomic structures of membranes [21].

**Table 1:** Program packages included in the CELLmicrocosmos 1.2 CellExplorer.

Name	Version	Usage	License	More info
Infonode Docking Windows	1.6.1	Manages the different windows in the main GUI	GPL	www.infonode.net
GlueGen	2.2.4	Call C libraries from java, used by JOGL	Simplified BSD 2	jogamp.org/gluegen/www/
j3d-core, vecmath	1.6.0	Displaying and picking of the 3D cell components	GPL 2 (with CLASSPATH exception)	forum.jogamp.org/Java3D-1-6-0-pre11-released-td4032735.html
j3d-core- utils	1.6.0	Picking and navigation	BSD (without advertising clause)	github.com/hharrison/java3d-utils
j3d-vrml97	0.1.0	VRML97 import	BSD	java.net/projects/j3d-vrml97
JDOM	1.1.1	Loading and saving of XML files	Apache License (without acknowledgment clause)	www.jdom.org
JOGL/JOAL	2.2.4	JogAmp’s java-binding for the OpenGL/OpenAL API	New BSD 2-clause license/simplified BSD 2	jogamp.org/jogl jogamp.org/joal
Substance	4.0	The L&F of the GUI	BSD (without advertising clause)	substance.dev.java.net

**Table 2:** The program packages included in the CELLmicrocosmos 4.2 PathwayIntegration module integrated in the CELLmicrocosmos 1.2 CellExplorer.

Name	Version	Usage	License	More info
jung- algorithmns	2.0	2D viewer	BSD License	jung.sourceforge.net
jung-api	2.0			
jung-graph- impl	2.0			
jung-io	2.0			
jungvisualiz ation	2.0			
collections- generic			Apache License 2.0	
axiom-api	1.2.7	Communication with databases via MySQL or web service connection	Apache License 2.0	
axiom-impl	1.2.7		Apache License 2.0	
axis-adb	1.4		Apache License 2.0	
axis-kernel	1.4		Apache License 2.0	
backport- util- concurrent	3.1		CPL/EPL	
commons- httpclient	3.1		Apache License 2.0	
commons- codec	3.1		Apache License 2.0	
commons- logging	11.1		Apache License 2.0	
concurrent			Public domain	g.oswego.edu/dl/class es/EDU/oswego/cs/dl/util/concurrent/intro.html
log4j	1.2.15		Apache License 2.0	
neethi	2.0.4		Apache License 2.0	
mysql- connector- java	5.15		GPL	
wsdl4j	1.6.2		CPL/EPL	
JFreeChart	1.0.13	Localization charts	LGPL 2.1 (or later)	www.jfree.org/jfreechart/
jSBML	1.0-rc1	SBML import and export	BSD License	sbml.org/Software/JSBML
Tools	from JDK 1.8.0_25	Compiling of java and jar files (node placing algorithms)	GPL 2 (with linking exception)	openjdk.java.net
XmlSchema	1.4.2	XML import and export	Apache License 2.0	

**Table 3:** Program packages included in the CELLmicrocosmos 2.2 MembraneEditor.

Name	Version	Usage	License	More info
Infonode Docking Windows	1.6.1	Manages the different windows in the main GUI	GPL	www.infonode.net
GlueGen	2.2.4	Call C libraries from Java, used by JOGL	Simplified BSD 2	jogamp.org/gluegen/www/
j3d-core, vecmath	1.6.0	Displaying and picking of the 3D membrane components	GPL 2 (with CLASSPATH exception)	forum.jogamp.org/Java3D-1-6-0-pre11-released-td4032735.html
j3d-core- utils	1.6.0	Picking and navigation	BSD (without advertising clause)	github.com/hharrison/java3d-utils
JDOM	1.1.1	Loading and saving of XML files	Apache License (without acknowledgment clause)	www.jdom.org
JFreeChart JCommon	1.0.13 1.0.16	Visualization of the resulting percentages lipid distributions and lipid/protein weights	LGPL 2.1 (or later)	www.jfree.org/jfreechart/
JOGL/JOAL	2.2.4	JogAmp’s Java-binding for the OpenGL/OpenAL API	New BSD 2-clause license/simplified BSD 2	jogamp.org/jogl jogamp.org/joal
Jmol	12.0.40	External previewing of the membrane or single molecules	LGPL 2.1 (or later)	jmol.sourceforge.net
Substance	4.0	The L&F of the GUI	BSD (without advertising clause)	substance.dev.java.net
Tools	from JDK 1.8.0_25	Compiling of java and jar files (membrane packing algorithms)	GPL 2 (with linking exception)	openjdk.java.net

In the beginning it was decided to use GPL 2 for the CELLmicrocosmos project, but as a number of used libraries were not compatible to GPL version 2, it was decided to use GPL version 3 which is more versatile.

## 5 Results and Discussion

The combination of Scientific and Information Visualization is a field which has currently large potential [44]. Both main tools presented here take both disciplines into account.

**Figure 8:**
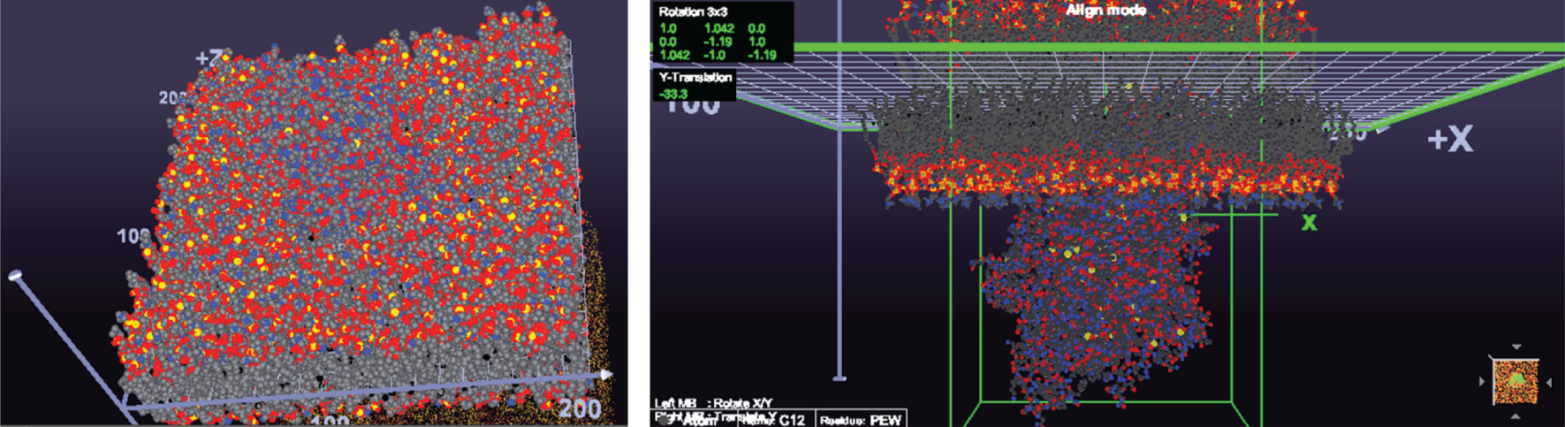
CELLmicrocosmos MembraneEditor: Two alternative ways to visualize the membrane: Left – Van der Waals visualization of the membrane surrounding a transmembrane protein – all atoms are visible, leading to a loss of overview and it is nearly impossible to differentiate between lipids; Right – the Align mode view of the transmembrane protein, showing only the protein and the neighboring lipids provides a better overview. Still, the diamond shape structure in Figure 5 enables the best differentiation between different molecules.

CmME provides different modes to visualize membranes. Figure 8 shows alternative ways to visualize the membrane shown in Figure 5: the Van der Waals visualization shows the atomic structure of all molecules (Figure 8 left), whereas the Align Mode shows only the transmembrane protein with the surrounding lipids (Figure 8 right). Obviously, the level of detail is much higher, whereas the differentiation of different structures is nearly impossible. Of course, by using, e.g. shading and ambient occlusion the visualization of the atomic structures could be drastically improved. However, still it will be nearly impossible to differentiate between different lipid molecules. The diamond-like visualization in Figure 5 enable an easy differentiation. While these approaches are more in the field of scientific visualization, the combination with the graphs showing the effective lipid distribution is an information graphic (Figure 5 right) as well as the table on the left showing the colour codes with the lipid distribution – the same colour codes are used for the colouring of the 3D lipids.

**Figure 9:**
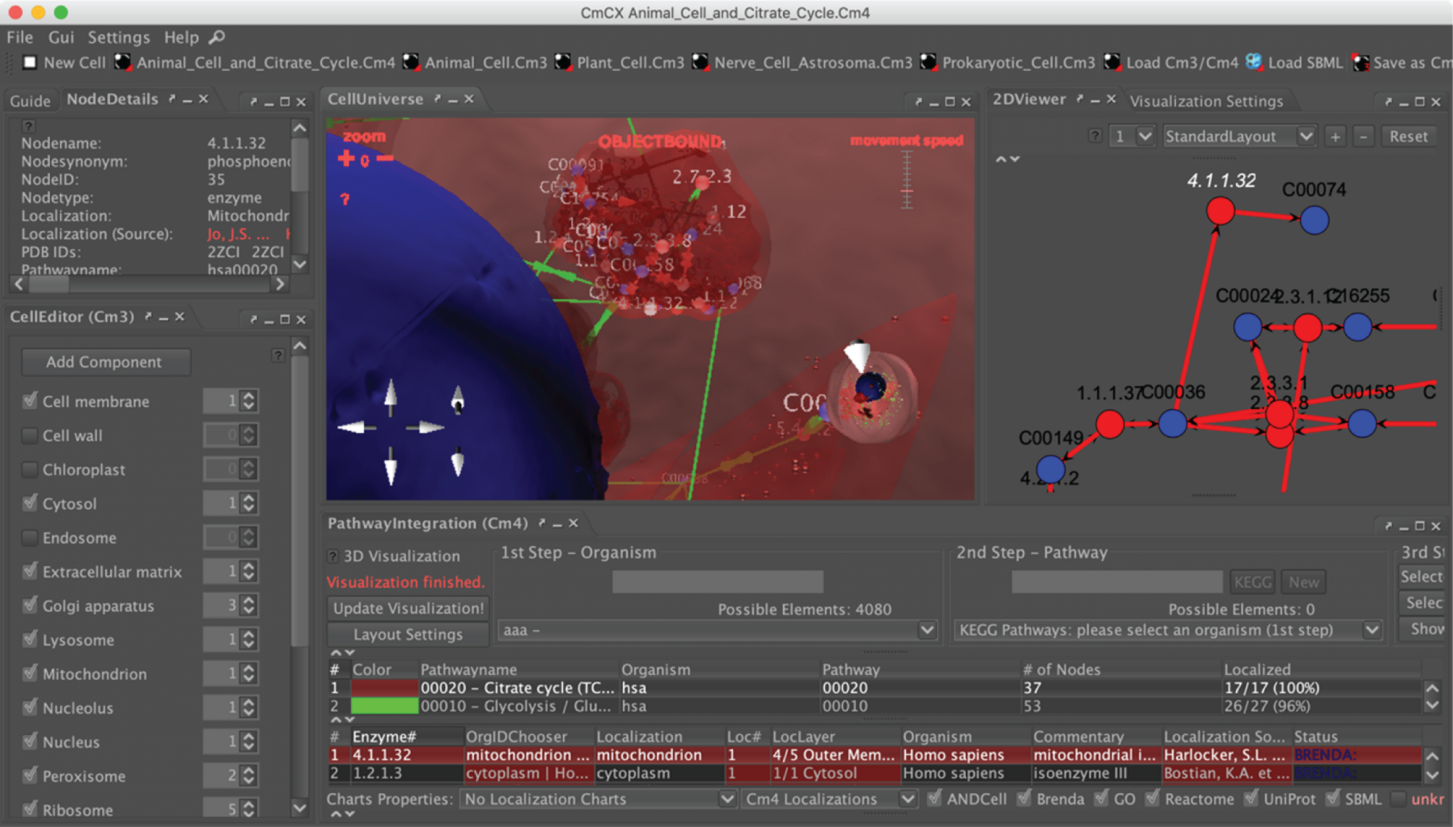
CELLmicrocosmos CellExplorer with the PathwayIntegration/Hybrid-dimensional visualization (a detail of Figure 6): Left – 3D visualization of the mitochondrion in the center associated with the KEGG citrate cycle; Right – 2D visualization of the same KEGG citrate cycle.

CmPI combines structural with information visualization: on one hand, the spatial structure of the cell components, and on the other one, the metabolic network. Figure 9 shows the internal structure of the cell, a detail of the cell shown in Figure 6: in the centre, the 3D structure of a mitochondrion is correlated with a metabolic network. While the 2D visualization is showing a classical KEGG-derived network, the 3D visualization embeds the network structure in the spatial context of the cell. The combination of 2D and 3D visualization is also known as hybrid-dimensional visualization [45].

Figure 1 showed already that a number of different student projects and theses lay the basis for this work. On one hand, students learn how to develop and realize new ideas and write new source code, on the other hand they have to learn to interpret the code of other students, improve and extend it. Of course, it is only possible to develop this large variety of projects over a long period of years if there is a main developer who is able to interpret and maintain the code. If the project leader is not able to do so, there must be funding for a software developer who can take over this task.

## 6 Outlook

Here, we discussed the two main CELLmicrocosmos tools supporting cell visualization and modelling on (1) the molecular level (with CmME), (2) mesoscopic (CmCX) and (3) functional level (CmPI) (see also Figure 2). An unreleased prototype implementation exists combining all these tools, merging these three levels into the integrative level (CmX) [2]. The extension of this prototype implementation might be a future direction merging all projects into one.

All discussed software approaches provide semi-immersive Virtual Reality-related visualization by supporting Stereoscopic 3D visualization. In case of CmCX/CmPI, if compatible hardware – such as the zSpace^®^ – is accessible, stereoscopic visualization, head tracking as well as 3D interaction is supported; this is important to provide Immersive Analytics capabilities [26], [46]. These approaches might be extended in the future to CmME.

While the CmME is used by a number of external projects, we are using it currently as part of a workflow to analyse antibiotics/membrane behaviour as part of a whole cell modelling project [47].

Moreover, certain aspects of the Cm project might be further developed into an educational direction.

As previously mentioned, the Cm tools run with Java 8. Therefore, the future use of new Java versions has to be evaluated, especially because access to future versions of Java will be restricted by Oracle and does not follow the vision of Open Source software development.
